# Utilizing Chimeric Antigen Receptors to Direct Natural Killer Cell Activity

**DOI:** 10.3389/fimmu.2015.00195

**Published:** 2015-04-28

**Authors:** David L. Hermanson, Dan S. Kaufman

**Affiliations:** ^1^Department of Medicine, Division of Hematology, Oncology, and Transplantation, University of Minnesota, Minneapolis, MN, USA; ^2^Stem Cell Institute, University of Minnesota, Minneapolis, MN, USA

**Keywords:** chimeric antigen receptors, natural killer cells, cancer immunotherapy, NK-92 cells, induced pluripotent stem cells

## Abstract

Natural killer (NK) cells represent an attractive lymphocyte population for cancer immunotherapy due to their ability to lyse tumor targets without prior sensitization and without need for human leukocyte antigens-matching. Chimeric antigen receptors (CARs) are able to enhance lymphocyte targeting and activation toward diverse malignancies. CARs consist of an external recognition domain (typically a small chain variable fragment) directed at a specific tumor antigen that is linked with one or more intracellular signaling domains that mediate lymphocyte activation. Most CAR studies have focused on their expression in T cells. However, use of CARs in NK cells is starting to gain traction because they provide a method to redirect these cells more specifically to target refractory cancers. CAR-mediated anti-tumor activity has been demonstrated using NK cell lines, as well as NK cells isolated from peripheral blood, and NK cells produced from human pluripotent stem cells. This review will outline the CAR constructs that have been reported in NK cells with a focus on comparing the use of different signaling domains in combination with other co-activating domains.

## Introduction

Natural killer (NK) cells are an important component of the innate immune system due to their ability to lyse infected or malignant cells without prior sensitization and without human leukocyte antigens (HLA)-restriction (1). They play an important role in immune surveillance and early control of many malignancies. NK cells recognize infected or transformed cells through multiple cell surface receptors including NKG2D, CD16 [the receptor that mediates antibody-dependent cellular cytotoxicity (ADCC)], and natural cytotoxicity receptors (NCRs) such as NKp44, NKp46, and NKp30 (2). These receptors activate signaling adapter proteins such as DAP10, DAP12, and CD3ζ, which contain immuno-tyrosine activation motifs (ITAMs) that initiate the release of cytolytic granules containing perforin and granzymes, as well as mediate production and release of cytokines and chemokines such as IFN-γ and TNF-α (3). Importantly, NK cell-mediated cytotoxicity does not rely on the presentation of self HLA. Therefore, NK cells hold significant clinical interest as a cell-based therapy for cancer because of their ability to be used in an allogeneic setting and potentially provide an off-the-shelf cellular product. Clinical trials using NK cells obtained from haploidentical donors demonstrate long-term remissions in patients with refractory acute myelogenous leukemia (4). Trials against solid tumors such as breast cancer and ovarian cancer have also demonstrated efficacy (5). NK cell lines (NK-92 cells) (6) and NK cells derived from umbilical cord blood (7) have also been tested in clinical trials (NCT01729091, NCT02280525).

Chimeric antigen receptors (CARs) are engineered proteins designed to activate lymphocytes, particularly T cells, upon target recognition. CARs contain a single-chain variable fragment (scFv) fused to a variety of possible intracellular signaling domain(s). The scFv is designed to target antigens either overexpressed or unique to tumor cells. The signaling domain initially tested was the ζ chain of the T cell receptor complex CD3 in first generation CARs (8). Second generation CARs employ co-activating proteins such as CD28, CD137 (4-1BB), or CD134 (OX40) in combination with CD3ζ to increase T cell activation and proliferation (9, 10). Finally, CAR constructs incorporate multiple co-activation domains and CD3ζ in the third generation CARs (11). The clinical success of anti-CD19 CAR-expressing T cells for treatment of B-cell malignancies has fueled the design and evaluation of CARs for T cell therapy toward other antigens and malignancies (12). While development of T cell-CAR-based therapies seems to be revolutionizing tumor immunotherapy, one major obstacle with this approach is the need to collect and utilize autologous cells. A second concern with the use of T cells is their long-term persistence, resulting in chronic on-target-off-tumor effects such as B-cell aplasia with the anti-CD19 CARs being used currently in clinical trials.

Natural killer cells provide an alternative to the use of T cells for adoptive immunotherapy since they do not require HLA matching, so can be used as allogeneic effector cells (13). Clinical trials of adoptively transferred allogeneic NK cells demonstrate these cells can survive in patients for several weeks to months (4, 13). Additionally, expression of CARs in NK cells may allow these cells to more effectively kill solid tumors that are often resistant to NK cell-mediated activity compared to hematologic malignancies (especially acute myelogenous leukemia) that are typically more NK cell-sensitive (4, 5). As such, CAR-expressing NK cells have gained significant interest to provide a targeted, allogeneic, “universal” cell population for treatment of refractory malignancies. This review will focus on the CAR constructs and activating domains that have been reported to be used in NK cell lines (such as NK-92 cells) and peripheral blood (PB) NK cells. Additional work using NK cells produced from human pluripotent stem cells is also discussed.

## Chimeric Antigen Receptors Used in NK Cell Lines

Natural killer cell lines have been utilized to evaluate CARs targeting several different antigens. By far, the most commonly studied NK cell line has been the NK-92 cell line, which has been previously (6) and is currently being used in clinical trials (NCT00900809 and NCT00990717). Other NK cell lines include NKG, YT, NK-YS, HANK-1, YTS cells, and NKL cells (14). Working with NK cell lines has several advantages such as providing a more homogeneous cell population compared to NK cells isolated from PB. In addition, the NK-92 cells have been well-defined and there is no need to perform any isolation of the NK cells from donors. However, NK cell lines also have distinct disadvantages. NK-92 cells lack the expression of several typical NK cell activating receptors such as CD16, NKp44, and NKp46 (15, 16). Also, NK-92 cells are tumor cell lines with multiple cytogenetic abnormalities (17) and are latently infected with Epstein–Barr virus (18). Therefore, for safety purposes, these cells must be irradiated prior to infusion.

The majority of studies to express CARs in NK-92 cells have used first generation CAR constructs that contain CD3ζ as their sole signaling domain. The scFvs of these CARs have targeted CD20 (19–21), CD19 (20–22), ErbB2 (HER2) (23–25), GD2 (26), and CD138 (27) (Table 1). In addition to directly targeting cell surface proteins, CARs can also recognize HLA-peptide complexes such as HLA-A2 expressing the melanoma-associated gp100 peptide (28). CARs directed toward CD19 and CD20 are designed to target B-cell malignancies and have also been studied extensively in T cells (29). The only other difference in the anti-CD19 or anti-CD20 CAR constructs used in NK-92 cells is the transmembrane region. One study used the CD3ζ transmembrane sequence (19) while another used the CD8 transmembrane sequence (22). However, without a direct comparison it is unknown if one construct is superior. Another study used an HLA-A2 transmembrane region coupled to a CD3ζ signaling domain (28), suggesting the transmembrane region may be easily altered without impacting CAR expression and functionality. Interestingly, comparison of CAR transfected NK-92 cells with ADCC function using NK-92 cells engineered to express CD16 found that the anti-CD20 CAR engineered cells lysed primary CLL cells more effectively than NK-92 cells acting through ADCC using rituximab (21). This study suggests that even first generation CARs may be an improvement over ADCC-mediated anti-tumor activity by NK-92 cells. It is important to note that NK-92 cells require transfection of CD16 in order to perform ADCC. This leaves open the possibility that PB-NK cells may still be better equipped to perform ADCC better than NK-92 cells.

**Table 1:** **CAR constructs utilized in NK cell lines (NK-92)**.

Reference	Target	CAR Construct	Method
(23)	ErbB2 (HER-2)	mCD8α hinge/CD3ζ	Amphotropic virus
(19)	CD20	mCD8α hinge/CD3ζ	Amphotropic virus
(22)	CD19	CD8α TM/CD3ζ	mRNA transfection (50%)
(30)	EpCAM	CD8α hinge/CD28/CD3ζ	Lentivirus along with IL-15
(31)	HLA-A2 EBNA3C	CD8α TM/CD137/CD3ζ	Retrovirus
(26)	GD2	mCD8α hinge/CD3ζ	Amphotropic virus
(20)	CD19/CD20	CD3ζ	mRNA transfection (30–70%) and lentivirus
(28)	HLA-2 complex with melanoma-associated gp100 peptide	A2 TM/CD3ζ	Transfection
(21)	CD19/CD20	CD3ζ	Lentivirus
(32)	CS1	CD28 TM/CD28/CD3ζ	Lentivirus
(27)	CD138	CD8α hinge/CD3ζ	Lentivirus
(24)	ErbB2 (HER-2)	CD8α hinge/CD28/CD3ζ	Transfection
(25)	ErbB2 (HER-2)	CD8α hinge/CD3ζ	Lentivirus
		CD8α hinge/CD28/CD3ζ	
		CD8α hinge/CD137/CD3ζ	
(34)	PSCA	CD28 hinge/CD28 TM/CD3ζ	Lentivirus in YTS NK cells and primary NK cell
		DAP12 TM and signaling	

Solid tumor antigens can also be targeted by first generation CAR constructs expressed in NK-92 cells. An anti-ErbB2 CAR construct against HER2-positive breast, ovarian, and squamous cell carcinoma cell lines mediated improved killing ability of NK-92 cells (23). Additionally, this study showed a reduction in tumor growth using ErbB2-expressing NIH 3.3 cells mixed with NK-92s in a subcutaneous mouse model (23). An anti-GD2 CAR using just the CD3ζ transmembrane and signaling domains was able to target primary glioblastoma cells as well as GD2-positive melanoma and breast carcinomas (26). NK-92 cells can also be targeted against multiple myeloma (MM) using an anti-CD138 CAR with only CD3ζ as a signaling domain (27). Notably, mice bearing a subcutaneous tumor treated with CAR-expressing NK-92 cells survived significantly longer than NK-92 cell alone in a CD138-positive tumor model; whereas, when a CD138-negative MM tumor was used no difference was detected (27). These data clearly demonstrate that first generation CARs are an effective means to induce target cell lysis in NK-92 cells both *in vitro* and in mouse models; however, many of the tumor models are subcutaneous, which may fail to properly recapitulate the complete tumor environment or NK cell trafficking issues.

Second generation CARs expressing a second signaling domain in conjunction with CD3ζ vastly improves the overall activity CAR-expressing T cells (9). This has generated interest in using second generation CARs in NK cells. Similar to first generation CARs, several different scFvs have been used with second generation CARs including EpCAM for multiple carcinomas including breast and ovarian cancer (30), an HLA-A2 EBNA3C complex for Epstein–Barr virus (31), CS1 for MM (32), and ErbB2 for HER2 positive cancers (24, 25). The most common second generation CAR utilized in NK-92 cells pairs the CD28 intracellular domain with CD3ζ (Table 1). Notably, NK cells do not naturally express CD28 (35); therefore, the effect that this domain has in NK cells is unclear. Other second generation CARs combine CD137 (4-1BB) intracellular domain with CD3ζ. Similar to first generation CARs, all of the constructs lead to antigen specific killing of target cells, displaying the diverse set of tumor antigens CARs can target. Comparison of an ErbB2 scFv fused with CD3ζ alone, CD28/CD3ζ, or CD137/CD3ζ tested head-to-head against breast cancer cells found that both of the second generation constructs improved killing compared to the first generation CARs (25). Specifically, the CD28/CD3ζ had 65% target lysis in ErbB2-positive MDA-MB453 while the CD137/CD3ζ lysed 62% and CD3ζ alone killed 51% (25). Another modification in their construct design was the modification of a cysteine to a serine in the CD8α signaling peptide used, which the authors suggest improves surface expression of the CAR in NK-92 cells. Finally, CD28/CD3ζ was compared to DAP12 alone using an anti-PSCA CAR in YTS NK cells for prostate cancer (34). In 293T cell lines engineered to express PSCA, a significant increase in cell killing was observed with the DAP12 containing CAR compared to the CD28/CD3ζ CAR, suggesting DAP12 may provide a better signaling domain than CD3ζ (34).

## Chimeric Antigen Receptor use in Peripheral Blood NK Cells

Chimeric antigen receptors have also been evaluated in PB-NK cells, which can be isolated from donors through simple blood draws or by apheresis if larger numbers of cells are needed. In contrast to NK-92 cells, activated PB-NK cells express a wider range of activating receptors, such as CD16, NKp44, and NKp46 as well as KIRs, which play an important role in NK cell licensing (36). In addition, PB-NK cells can be given without irradiating the cells so have the ability to expand *in vivo*, which has been correlated with effectiveness in trials involving AML (4). A greater variety of CAR constructs have been used and directly compared in PB-NK cells targeting CD19 (37–39), CD20 (33), or ErbB2 (40, 41) (Table 2). Imai et al. describe the use of two first generation anti-CD19 CARs, CD3ζ, or DAP10 signaling, and one second generation CAR with CD137 and CD3ζ. Compared to CD3ζ, DAP10 induced a much weaker response in PB-NK cells, and addition of the CD137 domain to the CAR resulted in augmented killing of RS4:11 and 380 (ALL) cell lines (37). Another study compared CD3ζ or 2B4 alone, 2B4 combined with CD3ζ, and a CD137/CD3ζ anti-CD19 CAR and tested them against the leukemic cell line REH. *In vitro* studies demonstrated the 2B4 alone CAR was slightly less active compared to CD3ζ alone. Comparing the second generation CARs, both were significantly better than CD3ζ alone while similar activity was observed in the 2B4/CD3ζ and CD137/CD3ζ CARs (38). When this work was extended to an anti-GD2 CAR for neuroblastoma with just the CD3ζ and 2B4/CD3ζ endodomains, again the 2B4/CD3ζ was significantly better than CD3ζ alone (38). Another study compared CD3ζ alone with a CD28/CD3ζ CAR using ErbB2 as a target. While no direct lysis experiment was performed, similar levels of INF-γ production were observed in PB-NK cells engineered with just CD3ζ or CD28/CD3ζ (41). While different measures were used, the finding that CD28/CD3ζ does not improve activity in PB-NK cells whereas the same construct was found to be more active in NK-92 suggests there may be differences in CAR activation of PB-NK and NK-92 cells.

**Table 2:** **CAR constructs utilized in PB-NK cells**.

Reference	Target	CAR construct	Method
(37)	CD19	CD8α TM/CD3ζ	Retrovirus (mean 69%)
		CD8α TM/DAP10	
		CD8α TM/CD137/CD3ζ	
(40)	ErbB2 (HER-2)	CD28/CD3ζ	Retrovirus (mean 55%)
(38)	CD19/GD2	CD3ζ or 2B4 alone	Retrovirus (13–24%)
		2B4/CD3ζ	
		CD8 TM/CD137/CD3ζ	
(39)	CD19	CD137/CD3ζ	Transfection (mRNA) (mean 58%)
(42)	NKG2D ligands	NKG2D/CD3ζ co-expressed with DAP10	Retrovirus and mRNA transfection
(41)	ErbB2 (HER-2)	CD3ζ alone	Retrovirus (40–50%)
		CD28/CD3ζ	
(33)	CD20	CD137/CD3ζ	Transfection (mRNA) (50–95%)

One unique approach to CAR creation was to use the ectodomain of NKG2D, an NK cell activation receptor, and link it directly to CD3ζ (42). This approach utilizes natural NKG2D ligands commonly overexpressed on malignant cells to active the CAR. Further, NKG2D associates with DAP10 providing a secondary signaling molecule. Indeed, co-expression of DAP10 with the NKG2D/CD3ζ CAR increased surface expression. This CAR was tested against multiple cell lines derived from several malignancies with the best responses demonstrated against ALL, osteosarcoma, prostate carcinoma, and rhabdomyosarcoma (42).

A third source of NK cells suitable for CAR expression are NK cells derived from human pluripotent stem cells – both induced pluripotent stem cells (iPSCs) or human embryonic stem cells (hESCs) (43–47). These NK cells display a similar phenotype to PB-NK cells (43, 44, 48), and hESC/iPSC-NK cells can be grown on a clinical scale (48). iPSC-derived NK cells engineered with a CD4/CD3ζ CAR are able to inhibit HIV replication (49). In these studies, the CAR was expressed in the iPSC cells, which were then differentiated into CAR-expressing iPSC-NK cells. The CD4/CD3ζ iPSC-NK cells were shown to suppress the *in vitro* replication of HIV, providing a platform from which to work for the further development of CAR positive iPSC-NK cells. iPSC-derived NK cells combine the best of PB-NK and NK-92 cells since the cells express NKp44, NKp46, and KIRs, are a homogeneous population with no evidence of undifferentiated iPSCs or T cells in the expanded NK cell population. Additionally, CARs can be easily expressed in hESC and/or iPSC-derived NK cells using non-viral gene transfer methods (49, 50). This is in contrast to PB-NK cells that are much more challenging to achieve high levels of stable CAR expression.

## Outlook

As the interest in using CARs in not only T cells (10) but also in NK cells continues to grow, there are still a number of questions that remain to be answered. Perhaps most important is what CAR constructs mediate optimal anti-tumor (or anti-viral) activity. Limited studies in NK-92 cells and in PB-NK cells directly compare first and second generation CARs. Second generation CARs in PB-NK cells are generally more active than first generation CARs. Additionally, the use of CD3ζ seems better than DAP10 as the signaling domain (37, 38). In NK-92 cells, DAP12 outperformed a CD28/CD3ζ CAR, but it remains unclear if NK-92 cells provide a good model for how CARs may function in PB-NK cells or hESC/iPSC-derived NK cells. Since NK cells do not naturally express CD28 (35, 51), it is not clear if CD28 is functioning in CAR-expressing NK cells. Different CAR constructs may be required to provide optimal NK cell activation depending on the tumor type or target antigen. More direct comparisons using various intracellular signaling domains and scFvs are needed to best resolve these questions.

Additional research is also needed to determine whether use of an NK cell line (such as NK-92 cells), PB-NK cells, or iPSC-NK cells will provide the best overall benefit. Both 4-1BBL/IL-15 (52) and mbIL-21 (53) artificial antigen presenting cells (aAPCs) can be used to expand PB-NK or iPSC-NK cells (48). Therefore, production of enough NK cells from these sources for clinical use is not a problem. However, it remains to be determined if one aAPC leads to an improved population for adoptive transfer, and the methods to engineer PB-NK cells still need to be further improved. iPSC-NK cells represent an attractive population of cells for NK-CAR therapy because once engineered the iPSC line can be maintained indefinitely and provide an almost limitless supply of NK cells. In addition, careful monitoring of the insertion site of the CAR can be achieved. Finally, NK cell lines provide another alternative but in general express fewer natural NK cell receptors and must be irradiated prior to infusion, which limits *in vivo* expansion and persistence of NK cells.

The method for CAR incorporation provides another important consideration. To get stable expression of CARs, retro- and lentivirus methods have dominated. However, following transduction of NK-92 cells a selection step is usually required to get a pure CAR-expressing population. In PB-NK cells, the efficiencies of gene transfer were at best 69% (37) and ranged as low as 13–24% (38) with most reporting around a 50% transduction efficiency. One way around this issue is the possibility of expressing the CAR in iPSCs and subsequent differentiation into mature NK cells (49), which is done via nucleofection with transposon and avoids the hazards of viral methods. Another consideration is whether the use of suicide systems, such as Cas9 or thymidine kinase (TK), will need to be put in place if unexpected toxicities arise despite the expectation that CAR-expressing NK cells will only circulate for a few weeks (14).

Despite the questions that remain, the ability to engineer NK cells with CARs holds great promise as a novel cellular immunotherapy against refractory malignancies and potentially chronic infectious diseases. The success of T cell-CARs in cases of ALL and CLL has revolutionized the prospects for cell-based immunotherapy. CAR-NK cells can build upon this success to provide important benefits as CAR-based therapy expands. Notably, NK cells can provide a homogenous, off-the-shelf, standardized product that can be used in as an allogeneic product to treat patients. Therefore, this process does not need to be done on a patient-specific basis, as with current T cell-CAR-based therapies. The ability to more potently direct NK cell-mediated cytotoxicity against refractory tumors through the expression of CARs can continue to revolutionize cancer treatment.

## Author Contributions

DH performed the review of the literature and wrote the manuscript. DK wrote and edited the manuscript.

## Conflict of Interest Statement

The authors declare that the research was conducted in the absence of any commercial or financial relationships that could be construed as a potential conflict of interest.

## References

[1] Vivier E Raulet DH Moretta A Caligiuri MA Zitvogel L Lanier LL Innate or adaptive immunity? The example of natural killer cells. Science (2011) 331:44–9. 10.1126/science.1198687 21212348 PMC3089969

[2] Long EO Kim HS Liu D Peterson ME Rajagopalan S. Controlling natural killer cell responses: integration of signals for activation and inhibition. Annu Rev Immunol (2013) 31:227–58. 10.1146/annurev-immunol-020711-075005 23516982 PMC3868343

[3] Lanier LL. Up on the tightrope: natural killer cell activation and inhibition. Nat Immunol (2008) 9:495–502. 10.1038/ni1581 18425106 PMC2669298

[4] Miller JS Soignier Y Panoskaltsis-Mortari A Mcnearney SA Yun GH Fautsch SK Successful adoptive transfer and in vivo expansion of human haploidentical NK cells in patients with cancer. Blood (2005) 105:3051–7. 10.1182/blood-2004-07-2974 15632206

[5] Geller MA Cooley S Judson PL Ghebre R Carson LF Argenta PA A phase II study of allogeneic natural killer cell therapy to treat patients with recurrent ovarian and breast cancer. Cytotherapy (2011) 13:98–107. 10.3109/14653249.2010.515582 20849361 PMC3760671

[6] Tonn T Schwabe D Klingemann HG Becker S Esser R Koehl U Treatment of patients with advanced cancer with the natural killer cell line NK-92. Cytotherapy (2013) 15:1563–70. 10.1016/j.jcyt.2013.06.017 24094496

[7] Spanholtz J Preijers F Tordoir M Trilsbeek C Paardekooper J De Witte T Clinical-grade generation of active NK cells from cord blood hematopoietic progenitor cells for immunotherapy using a closed-system culture process. PLoS One (2011) 6:e20740. 10.1371/journal.pone.0020740 21698239 PMC3116834

[8] Gross G Waks T Eshhar Z. Expression of immunoglobulin-T-cell receptor chimeric molecules as functional receptors with antibody-type specificity. Proc Natl Acad Sci U S A (1989) 86:10024–8. 10.1073/pnas.86.24.10024 2513569 PMC298636

[9] Sadelain M Brentjens R Rivière I. The basic principles of chimeric antigen receptor design. Cancer Discov (2013) 3:388–98. 10.1158/2159-8290.CD-12-0548 23550147 PMC3667586

[10] Jena B Moyes JS Huls H Cooper LJ. Driving CAR-based T-cell therapy to success. Curr Hematol Malig Rep (2014) 9:50–6. 10.1007/s11899-013-0197-7 24488441 PMC3995024

[11] Carpenito C Milone MC Hassan R Simonet JC Lakhal M Suhoski MM Control of large, established tumor xenografts with genetically retargeted human T cells containing CD28 and CD137 domains. Proc Natl Acad Sci U S A (2009) 106:3360–5. 10.1073/pnas.0813101106 19211796 PMC2651342

[12] Kakarla S Gottschalk S. CAR T cells for solid tumors: armed and ready to go? Cancer J (2014) 20:151–5. 10.1097/PPO.0000000000000032 24667962 PMC4050065

[13] Vivier E Tomasello E Baratin M Walzer T Ugolini S Functions of natural killer cells. Nat Immunol (2008) 9:503–10 10.1038/ni1582 18425107

[14] Glienke W Esser R Priesner C Suerth JD Schambach A Wels WS Advantages and applications of CAR-expressing natural killer cells. Front Pharmacol (2015) 6:21. 10.3389/fphar.2015.00021 25729364 PMC4325659

[15] Gong JH Maki G Klingemann HG. Characterization of a human cell line (NK-92) with phenotypical and functional characteristics of activated natural killer cells. Leukemia (1994) 8:652–8. 8152260

[16] Maki G Klingemann HG Martinson JA Tam YK. Factors regulating the cytotoxic activity of the human natural killer cell line, NK-92. J Hematother Stem Cell Res (2001) 10:369–83. 10.1089/152581601750288975 11454312

[17] MacLeod RA Nagel S Kaufmann M Greulich-Bode K Drexler HG. Multicolor-FISH analysis of a natural killer cell line (NK-92). Leuk Res (2002) 26:1027–33. 10.1016/S0145-2126(02)00055-3 12363472

[18] Uphoff CC Denkmann SA Steube KG Drexler HG. Detection of EBV, HBV, HCV, HIV-1, HTLV-I and -II, and SMRV in human and other primate cell lines. J Biomed Biotechnol (2010) 2010:904767. 10.1155/2010/904767 20454443 PMC2861168

[19] Müller T Uherek C Maki G Chow KU Schimpf A Klingemann HG Expression of a CD20-specific chimeric antigen receptor enhances cytotoxic activity of NK cells and overcomes NK-resistance of lymphoma and leukemia cells. Cancer Immunol Immunother (2008) 57:411–23. 10.1007/s00262-007-0383-3 17717662 PMC11029838

[20] Boissel L Betancur M Lu W Wels WS Marino T Van Etten RA Comparison of mRNA and lentiviral based transfection of natural killer cells with chimeric antigen receptors recognizing lymphoid antigens. Leuk Lymphoma (2012) 53:958–65. 10.3109/10428194.2011.634048 22023526 PMC3491067

[21] Boissel L Betancur-Boissel M Lu W Krause DS Van Etten RA Wels WS Retargeting NK-92 cells by means of CD19- and CD20-specific chimeric antigen receptors compares favorably with antibody-dependent cellular cytotoxicity. Oncoimmunology (2013) 2:e26527. 10.4161/onci.26527 24404423 PMC3881109

[22] Boissel L Betancur M Wels WS Tuncer H Klingemann H. Transfection with mRNA for CD19 specific chimeric antigen receptor restores NK cell mediated killing of CLL cells. Leuk Res (2009) 33:1255–9. 10.1016/j.leukres.2008.11.024 19147228 PMC3047414

[23] Uherek C Tonn T Uherek B Becker S Schnierle B Klingemann HG Retargeting of natural killer-cell cytolytic activity to ErbB2-expressing cancer cells results in efficient and selective tumor cell destruction. Blood (2002) 100:1265–73. 12149207

[24] Liu H Yang B Sun T Lin L Hu Y Deng M Specific growth inhibition of ErbB2-expressing human breast cancer cells by genetically modified NK-92 cells. Oncol Rep (2015) 33:95–102. 10.3892/or.2014.3548 25333815

[25] Schönfeld K Sahm C Zhang C Naundorf S Brendel C Odendahl M Selective inhibition of tumor growth by clonal NK cells expressing an ErbB2/HER2-specific chimeric antigen receptor. Mol Ther (2015) 23:330–8. 10.1038/mt.2014.219 25373520 PMC4445620

[26] Esser R Müller T Stefes D Kloess S Seidel D Gillies SD NK cells engineered to express a GD2-specific antigen receptor display built-in ADCC-like activity against tumour cells of neuroectodermal origin. J Cell Mol Med (2012) 16:569–81. 10.1111/j.1582-4934.2011.01343.x 21595822 PMC3822932

[27] Jiang H Zhang W Shang P Zhang H Fu W Ye F Transfection of chimeric anti-CD138 gene enhances natural killer cell activation and killing of multiple myeloma cells. Mol Oncol (2014) 8:297–310. 10.1016/j.molonc.2013.12.001 24388357 PMC5528539

[28] Zhang G Liu R Zhu X Wang L Ma J Han H Retargeting NK-92 for anti-melanoma activity by a TCR-like single-domain antibody. Immunol Cell Biol (2013) 91:615–24. 10.1038/icb.2013.45 24100387

[29] Ghorashian S Pule M Amrolia P CD19 chimeric antigen receptor T cell therapy for haematological malignancies. Br J Haematol (2015). 10.1111/bjh.13340 25753571

[30] Sahm C Schönfeld K Wels WS. Expression of IL-15 in NK cells results in rapid enrichment and selective cytotoxicity of gene-modified effectors that carry a tumor-specific antigen receptor. Cancer Immunol Immunother (2012) 61:1451–61. 10.1007/s00262-012-1212-x 22310931 PMC11029748

[31] Tassev DV Cheng M Cheung NK. Retargeting NK92 cells using an HLA-A2-restricted, EBNA3C-specific chimeric antigen receptor. Cancer Gene Ther (2012) 19:84–100. 10.1038/cgt.2011.66 21979579

[32] Chu J Deng Y Benson DM He S Hughes T Zhang J CS1-specific chimeric antigen receptor (CAR)-engineered natural killer cells enhance in vitro and in vivo antitumor activity against human multiple myeloma. Leukemia (2014) 28:917–27. 10.1038/leu.2013.279 24067492 PMC3967004

[33] Chu Y Hochberg J Yahr A Ayello J Van De Ven C Barth M Targeting CD20+ aggressive B-cell Non-Hodgkin lymphoma by anti-CD20 CAR mRNA-modified expanded natural killer cells in vitro and in NSG mice. Cancer Immunol Res (2015) 3(4):333–44. 10.1158/2326-6066.CIR-14-0114 25492700

[34] Töpfer K Cartellieri M Michen S Wiedemuth R Müller N Lindemann D DAP12-based activating chimeric antigen receptor for NK cell tumor immunotherapy. J Immunol (2015) 194(7):3201–12. 10.4049/jimmunol.1400330 25740942

[35] Lang S Vujanovic NL Wollenberg B Whiteside TL. Absence of B7.1-CD28/CTLA-4-mediated co-stimulation in human NK cells. Eur J Immunol (1998) 28:780–6. 10.1002/(SICI)1521-4141(199803)28:03<780::AID-IMMU780>3.3.CO;2-# 9541571

[36] Kim S Poursine-Laurent J Truscott SM Lybarger L Song YJ Yang L Licensing of natural killer cells by host major histocompatibility complex class I molecules. Nature (2005) 436:709–13. 10.1038/nature03847 16079848

[37] Imai C Iwamoto S Campana D. Genetic modification of primary natural killer cells overcomes inhibitory signals and induces specific killing of leukemic cells. Blood (2005) 106:376–83. 10.1182/blood-2004-12-4797 15755898 PMC1895123

[38] Altvater B Landmeier S Pscherer S Temme J Schweer K Kailayangiri S 2B4 (CD244) signaling by recombinant antigen-specific chimeric receptors costimulates natural killer cell activation to leukemia and neuroblastoma cells. Clin Cancer Res (2009) 15:4857–66. 10.1158/1078-0432.CCR-08-2810 19638467 PMC2771629

[39] Li L Liu LN Feller S Allen C Shivakumar R Fratantoni J Expression of chimeric antigen receptors in natural killer cells with a regulatory-compliant non-viral method. Cancer Gene Ther (2010) 17:147–54. 10.1038/cgt.2009.61 19745843 PMC2821468

[40] Kruschinski A Moosmann A Poschke I Norell H Chmielewski M Seliger B Engineering antigen-specific primary human NK cells against HER-2 positive carcinomas. Proc Natl Acad Sci U S A (2008) 105:17481–6. 10.1073/pnas.0804788105 18987320 PMC2582261

[41] Alsamah W Romia Y Modification of natural killer cells to target tumors. Int J Pharm Clin Res (2014) 6(1):97–100.

[42] Chang YH Connolly J Shimasaki N Mimura K Kono K Campana D. A chimeric receptor with NKG2D specificity enhances natural killer cell activation and killing of tumor cells. Cancer Res (2013) 73:1777–86. 10.1158/0008-5472.CAN-12-3558 23302231

[43] Woll PS Martin CH Miller JS Kaufman DS. Human embryonic stem cell-derived NK cells acquire functional receptors and cytolytic activity. J Immunol (2005) 175:5095–103. 10.4049/jimmunol.175.8.5095 16210613

[44] Woll PS Grzywacz B Tian X Marcus RK Knorr DA Verneris MR Human embryonic stem cells differentiate into a homogeneous population of natural killer cells with potent in vivo antitumor activity. Blood (2009) 113:6094–101. 10.1182/blood-2008-06-165225 19365083 PMC2699231

[45] Ni Z Knorr DA Clouser CL Hexum MK Southern P Mansky LM Human pluripotent stem cells produce natural killer cells that mediate anti-HIV-1 activity by utilizing diverse cellular mechanisms. J Virol (2011) 85:43–50. 10.1128/JVI.01774-10 20962093 PMC3014194

[46] Ni Z Knorr DA Kaufman DS. Hematopoietic and nature killer cell development from human pluripotent stem cells. Methods Mol Biol (2013) 1029:33–41. 10.1007/978-1-62703-478-4_3 23756940

[47] Eguizabal C Zenarruzabeitia O Monge J Santos S Vesga MA Maruri N Natural killer cells for cancer immunotherapy: pluripotent stem cells-derived NK cells as an immunotherapeutic perspective. Front Immunol (2014) 5:439. 10.3389/fimmu.2014.00439 25309538 PMC4164009

[48] Knorr DA Ni Z Hermanson D Hexum MK Bendzick L Cooper LJ Clinical-scale derivation of natural killer cells from human pluripotent stem cells for cancer therapy. Stem Cells Transl Med (2013) 2:274–83. 10.5966/sctm.2012-0084 23515118 PMC3659832

[49] Ni Z Knorr DA Bendzick L Allred J Kaufman DS. Expression of chimeric receptor CD4ζ by natural killer cells derived from human pluripotent stem cells improves in vitro activity but does not enhance suppression of HIV infection in vivo. Stem Cells (2014) 32:1021–31. 10.1002/stem.1611 24307574 PMC3960346

[50] Wilber A Linehan JL Tian X Woll PS Morris JK Belur LR Efficient and stable transgene expression in human embryonic stem cells using transposon-mediated gene transfer. Stem Cells (2007) 25:2919–27. 10.1634/stemcells.2007-0026 17673526

[51] Goodier MR Londei M. CD28 is not directly involved in the response of human CD3- CD56+ natural killer cells to lipopolysaccharide: a role for T cells. Immunology (2004) 111:384–90. 10.1111/j.0019-2805.2004.01834.x 15056374 PMC1782433

[52] Fujisaki H Kakuda H Shimasaki N Imai C Ma J Lockey T Expansion of highly cytotoxic human natural killer cells for cancer cell therapy. Cancer Res (2009) 69:4010–7. 10.1158/0008-5472.CAN-08-3712 19383914 PMC2716664

[53] Denman CJ Senyukov VV Somanchi SS Phatarpekar PV Kopp LM Johnson JL Membrane-bound IL-21 promotes sustained ex vivo proliferation of human natural killer cells. PLoS One (2012) 7:e30264. 10.1371/journal.pone.0030264 22279576 PMC3261192

